# Sustained Hypoxia‐Inducible Factor 1‐Alpha Accumulation Disrupts the Articular Niche to Promote Osteoarthritis Pathogenesis

**DOI:** 10.1111/acel.70606

**Published:** 2026-06-23

**Authors:** Weiyuan Gong, Chu Tao, Xingyun Wang, Rongdong Liao, Jianglong Li, Xiongtian Guo, Minghao Qu, Jianmei Huang, Mingjue Chen, Fuxin Wei, Peng Wang, Lijun Lin, Di Chen, Qing Yao, Chunyi Wen, Guozhi Xiao

**Affiliations:** ^1^ Department of Biomedical Engineering The Hong Kong Polytechnic University Hong Kong China; ^2^ Department of Biochemistry, School of Medicine, Guangdong Provincial Key Laboratory of Cell Microenvironment and Disease Research, Shenzhen Key Laboratory of Cell Microenvironment Southern University of Science and Technology Shenzhen China; ^3^ Shenzhen Key Laboratory of Bone Tissue Repair and Translational Research, Department of Orthopaedic Surgery The Seventh Affiliated Hospital of Sun Yat‐Sen University Shenzhen China; ^4^ Department of Pharmacology, School of Medicine Southern University of Science and Technology Shenzhen China; ^5^ Sichuan Provincial People's Hospital Sichuan Academy of Medical Sciences Chengdu China; ^6^ Department of Joint and Orthopedics Zhujiang Hospital, Southern Medical University Guangzhou China; ^7^ Faculty of Pharmaceutical Sciences Shenzhen Institute of Advanced Technology Shenzhen China

**Keywords:** cartilage, HIF‐1α, LNP‐mRNA, OA

## Abstract

The precise role of hypoxia‐inducible factor‐1α (HIF‐1α) in osteoarthritis (OA) pathogenesis remains controversial, often debated between a protective compensatory factor and a disease mediator. Here, we demonstrate that sustained, uncoupled HIF‐1α accumulation functions as a potent, compartment‐specific pathogenic driver of joint destruction. Using genetically engineered mouse models, we reveal that chondrocyte‐specific HIF‐1α overexpression (*Acan* ^*CreERT2*^; *Hif1αdPA* ^*fl/fl*^) triggers spontaneous OA and exacerbates destabilization of the medial meniscus (DMM)‐induced post‐traumatic joint degeneration. Mechanistically, continuous HIF‐1α activation drives pathological angiogenesis that physically dismantles the avascular, hypoxic cartilage niche, forcing a profound metabolic dysregulation that culminates in catastrophic matrix degradation. Conversely, sustained HIF‐1α activation within the synovial and superficial cartilage compartments (*Prg4‐ GFPCreERT2*; *Hif1αdPA* ^*fl/fl*^) drives a slowly progressive, late‐onset spontaneous OA through chronic inflammatory accumulation that actively suppresses Col2a1 expression. Furthermore, this robust inflammatory priming establishes a highly vulnerable microenvironment, whereby DMM surgery significantly accelerates the progression of trauma‐induced joint collapse. Finally, transient whole‐joint HIF‐1α induction via an intra‐articular injection of lipid nanoparticles (LNP‐mRNA) closely recapitulates these detrimental effects. Collectively, our study reconciles existing controversies by establishing sustained HIF‐1α accumulation as a spatiotemporally dynamic, broad disease amplifier across the articular ecosystem, highlighting its targeted inhibition as a promising therapeutic strategy for OA.

## Graphical abstract

Sustained HIF‐1α accumulation is not a compensatory shield, but a potent pathogenic driver of osteoarthritis. Continuous HIF‐1α signaling physically eradicates the avascular cartilage niche and primes the synovium for inflammation, establishing HIF‐1α as a critical target for disease‐modifying therapies.

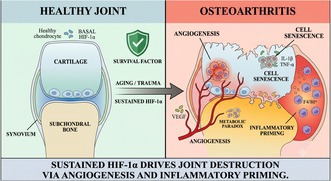

## 1 Introduction

Osteoarthritis (OA) represents a major global health burden, characterized by progressive damage to articular cartilage, reactive hyperplasia at the joint margins, and pathological changes in the subchondral bone (Hunter 2011; Chen et al. 2025, 2026). While traditionally viewed as a passive “wear and tear” disease, OA is now recognized as a metabolically active process involving complex interactions between mechanical stress, inflammatory mediators, and cellular metabolic dysregulation (Mobasheri et al. 2017; Wei et al. 2023; Welhaven et al. 2024). Despite its massive socioeconomic burden, current pharmacological interventions remain largely palliative (Karsdal et al. 2016). This critical translational gap primarily stems from an incomplete understanding of the precise molecular drivers that dynamically disrupt this whole‐joint ecosystem.

A hallmark of healthy articular cartilage is a highly specialized, avascular, aneural and hypoxic microenvironment. To survive and function within this extreme niche, chondrocytes rely heavily on hypoxia‐inducible factor‐1α (HIF‐1α), the master transcriptional regulator of the cellular hypoxic response. Under physiological conditions, HIF‐1α acts as an essential survival factor, driving anaerobic glycolysis and supporting extracellular matrix (ECM) anabolism to maintain cartilage integrity during skeletal development and adult homeostasis (Sophia Fox et al. 2009; Kuyinu et al. 2016).

In cartilage biology, HIF‐1α plays a paradoxical role. Early developmental studies established HIF‐1α as an essential survival factor for maintaining chondrocyte viability and energy metabolism. Paradoxically, however, extensive clinical and preclinical evidence indicates that HIF‐1α expression is abnormally and chronically upregulated in the cartilage and synovium of osteoarthritic joints (Qing et al. 2016). This observation has prompted a long‐standing debate regarding its precise pathogenic role (Taheem et al. 2020). Indeed, some studies suggest that genetic deletion of *Hif1α* accelerates cartilage destruction (Bouaziz et al. 2016), framing its upregulation in OA as an unsuccessful compensatory attempt to rescue the damaged joint. Conversely, accumulating evidence implicates aberrant hypoxia‐inducible signaling in driving pathological angiogenesis and inflammatory cascades. Unlike physiological hypoxia, chronic HIF‐1α accumulation is hypothesized to induce pro‐angiogenic factors (e.g., vascular endothelial growth factor [VEGF]) that physically dismantle the articular ecosystem by promoting vascular invasion into the strictly avascular cartilage niche (Pfander et al. 2003; Mapp and Walsh 2012; Caliogna et al. 2024). Whether the sustained, uncoupled accumulation of HIF‐1α itself acts as a protective shield or a pathogenic amplifier remains highly controversial.

These conflicting perspectives largely stem from fundamental limitations in previous studies: First, the reliance on global interventions or noninducible genetic models that fail to capture the spatiotemporal dynamics of the disease. Second, previous efforts to investigate HIF‐1α accumulation have primarily relied on the indirect stabilization of HIFs via the genetic deletion of upstream negative regulators, such as *Vhl* or *Phd2* (Cheng et al. 2017; Weng et al. 2014). Because VHL and PHDs act as broad oxygen sensors that regulate multiple downstream targets (including HIF‐2α and other non‐HIF pathways), these indirect approaches inevitably introduce confounding off‐target effects that obscure the specific pathogenic contribution of HIF‐1α itself. The joint ecosystem comprises distinct functional niches with vastly different baseline oxygen tensions, cell origins, and vascularity (Roelofs et al. 2017). The deep‐zone cartilage relies on strict avascularity, whereas the *Prg4*‐expressing superficial cartilage and synovial compartments are in close proximity to the vascular network, serving as critical hubs for joint lubrication and inflammatory responses (Lefebvre and Bhattaram 2015; Rhee et al. 2005; Mathiessen and Conaghan 2017; Sellam and Berenbaum 2010). How chronic HIF‐1α accumulation independently disrupts these distinct microenvironments, specifically regarding pathological angiogenesis, inflammatory priming, and cellular matrix metabolism, remains largely unknown.

In this study, we hypothesize that uncoupled, continuous HIF‐1α accumulation functions not as a static protector, but as a potent, context‐dependent pathogenic amplifier. To rigorously test this, we utilized temporally controlled, compartment‐specific in vivo genetic models (*Acan* ^*CreERT2*^ and *Prg4* ^*GFPCreERT2*^) (Yao, Gong, et al. 2023), combined with a degradation‐resistant HIF‐1α mutant (*Hif1αdPA* ^*fl/fl*^). We demonstrate that deep‐zone chondrocyte HIF‐1α overexpression intrinsically drives a severe cellular metabolic dysregulation. Concurrently, it induces pathological angiogenesis that physically eradicates the essential hypoxic cartilage niche, synergistically accelerating joint collapse. Furthermore, we reveal a novel mechanism whereby sustained HIF‐1α activation within the *Prg4*‐expressing superficial niche drives late‐onset OA by establishing a chronic state of “inflammatory priming and anabolic suppression.” This microenvironmental deterioration actively suppresses Col2a1 expression without immediately triggering catabolic surges, thereby lowering the threshold for trauma‐induced joint collapse. Finally, using an intra‐articular injection of lipid nanoparticles (LNP‐mRNA) (Mitchell et al. 2021; Zong et al. 2023), we validate that transient, whole‐joint HIF‐1α induction rigorously phenocopies these destructive effects. Collectively, our findings reconcile existing controversies by establishing sustained HIF‐1α activation as a broad driver of whole‐joint deterioration, unveiling a crucial therapeutic target for OA intervention.

## 2 Materials and Methods

### 2.1 Human Samples

Human articular cartilage and synovial tissues were obtained from a single patient diagnosed with advanced OA who was hospitalized for total joint replacement surgeries and were recruited in this study. The collection and experimental use of the clinical specimens were approved by the Institutional Ethics Committee of the Seventh Affiliated Hospital of Sun Yat‐sen University (Approval No. KY‐2026‐178‐01), and written informed consent was obtained from the patient. Several macroscopically degraded osteochondral regions and inflamed synovial tissues were collected as the OA group. Simultaneously, multiple macroscopically intact cartilage regions and adjacent noninflamed synovial tissues were isolated from the same joint to serve as intra‐patient controls. Tissues were fixed in 4% paraformaldehyde. Osteochondral samples were further decalcified in 15% EDTA (Wu, Lai, et al. 2022). All specimens were embedded in paraffin and sectioned for further histological and IF analysis.

### 2.2 Animals

*Prg4* ^*GFPCreERT2*^ and *Hif1αdPA* ^*fl/fl*^ mice were purchased from Jackson Laboratory. The *Hif1αdPA* ^*fl/fl*^ mice harbor a human cDNA encoding the HIF‐1α mutant protein inserted into the ROSA26 locus and preceded by a stop cassette flanked by LoxP sites. This mutant protein cannot be hydroxylated by the oxygen sensors HIF prolyl‐4‐hydroxylases (PHDs) 1/2/3, as the two prolines that are the targets of the PHDs have been replaced with alanines. Consequently, the HIF1αdPA mutant protein evades proteasomal degradation, leading to the constant activation of HIF1 signaling irrespective of oxygen levels. The transcriptional characteristics of this mutant protein are identical to those of wild‐type HIF‐1α. *Aggrecan* (*Acan*)^*CreERT2*^ and *Prg4* ^*GFPCreERT2*^ were described previously (Yao, Gong, et al. 2023; Wu, Lai, et al. 2022; Gan et al. 2024). We bred *Hif1αdPA* ^*fl/fl*^ mice with *Acan* ^*CreERT2*^ mice to obtain *Acan* ^*CreERT2*^; *Hif1αdPA* ^*fl/+*^ mice. Then, the *Hif1αdPA* ^*fl/fl*^ mice were mated with *Acan* ^*CreERT2*^; *Hif1αdPA* ^*fl/+*^ mice to obtain the required *Acan* ^*CreERT2*^; *Hif1αdPA* ^*fl/fl*^ mice for the experiment (Figure S1). *Prg4* ^*GFPCreERT2*^; *Hif1αdPA* ^*fl/fl*^ mice were generated using a similar breeding strategy. All research protocols in this study were approved by the Institutional Animal Care and Use Committees (IACUC) of Southern University of Science and Technology (SUSTech‐SL2025090803).

### 2.3 Destabilization of the Medial Meniscus (DMM) Surgery

Following isoflurane anesthesia, the right knee joints of the mice were shaved and prepared aseptically. A medial parapatellar incision was utilized to access the joint capsule. Osteoarthritis was surgically induced by transecting the medial meniscotibial ligament with micro‐scissors to create joint instability, carefully preserving the meniscus. The joint capsule and skin were subsequently sutured. Sham surgeries, consisting of arthrotomy without ligament transection, were performed on the contralateral left knees. Postoperative analgesia was administered for 48 h (Glasson et al. 2007).

### 2.4 Micro‐Computed Tomography (μCT) Analysis

According to the protocol, knee joints were scanned using a high‐resolution micro‐CT system (Bruker Skyscan 1276) at 60 kV and 100 μA. Scans were performed at an isotropic voxel size of 13 μm for live mice and 10 μm for formalin‐fixed specimens (Wu, Lai, et al. 2022; Wu, Qu, et al. 2022). All reconstructions were conducted using consistent thresholding parameters to enable three‐dimensional structural rendering and analysis.

### 2.5 Histology and Immunostaining Assays

Mouse knee joints were fixed in 4% paraformaldehyde (PFA) for 24 h at 4°C, followed by decalcification in 10% EDTA (pH 7.2) for 21 days. The tissues were then dehydrated through a graded ethanol series, cleared in xylene, and embedded in paraffin (Wu, Lai, et al. 2022; Chen et al. 2022). Serial sections (5 μm thickness) were prepared and stained with Hematoxylin and Eosin (H&E; Thermo Fisher, #7211 & 7111) or Safranin O and Fast Green (SO&FG; Solarbio, #G1371) according to the manufacturers' protocols for general morphological and cartilage‐specific evaluation (Wu, Lai, et al. 2022; Chaplan et al. 1994; Tao et al. 2024).

For IF staining, tissue sections underwent the same initial preparation steps as for histological analysis, including deparaffinization, rehydration, and antigen retrieval. After antigen retrieval, sections were permeabilized with 0.1% Triton X‐100 in PBS for 15 min and then blocked for 1 h at room temperature using a commercial blocking buffer (QuickBlock from Beyotime) to prevent nonspecific antibody binding. The sections were then incubated with primary antibodies diluted in the blocking buffer at 4°C overnight. Following extensive washes, the sections were incubated with species‐specific secondary antibodies conjugated to Alexa Fluor 568, with all steps performed in the dark to prevent fluorophore bleaching. Cell nuclei were counterstained with DAPI (1 μg/mL) for 5–10 min. After a final series of washes, the sections were mounted using an anti‐fade mounting medium (Lai et al. 2022; Gao et al. 2022; Yan et al. 2026). Stained sections were examined and imaged using a ZEISS LSM 980 confocal microscope. Image analysis and fluorescence intensity quantification were performed using ImageJ software. All histological scoring and quantitative analyses for IF were conducted in a double‐blinded manner to ensure objectivity.

### 2.6 Animal Behavioral Tests

Mechanical allodynia was assessed using the von Frey filament test, following an established protocol with minor modifications. Mice were acclimatized for 15 min on an elevated mesh platform prior to testing. A series of calibrated von Frey filaments (Stoelting) were applied perpendicularly to the plantar surface of the hind paw with sufficient force to cause slight bending. The 50% mechanical withdrawal threshold was determined using the up‐down method (Chaplan et al. 1994; Bonin et al. 2014). All tests were conducted by an investigator blinded to the experimental groups to minimize bias.

### 2.7 Statistics

The sample size for each experiment was determined based on preliminary data from our laboratory's previous animal studies and power analysis, ensuring adequate statistical power to detect anticipated effect sizes. This study has been conducted with anonymized data and analysis procedures in accordance with the dual‐blind statistical analysis requirements. All statistical analyses were performed using GraphPad Prism software (Graph Pad Prism 8.0). Data are presented as the mean ± standard deviation (SD). For comparisons between two independent groups, a two‐sided unpaired Student's *t*‐test was applied, assuming data normality and homogeneity of variance. A *p*‐value of less than 0.05 was considered statistically significant. All experiments were repeated at least three times.

## 3 Results

### 3.1 Expression Patterns of HIF‐1α and Vegf Are Altered in Age‐Related and Post‐Traumatic Osteoarthritis

**FIGURE 1:**
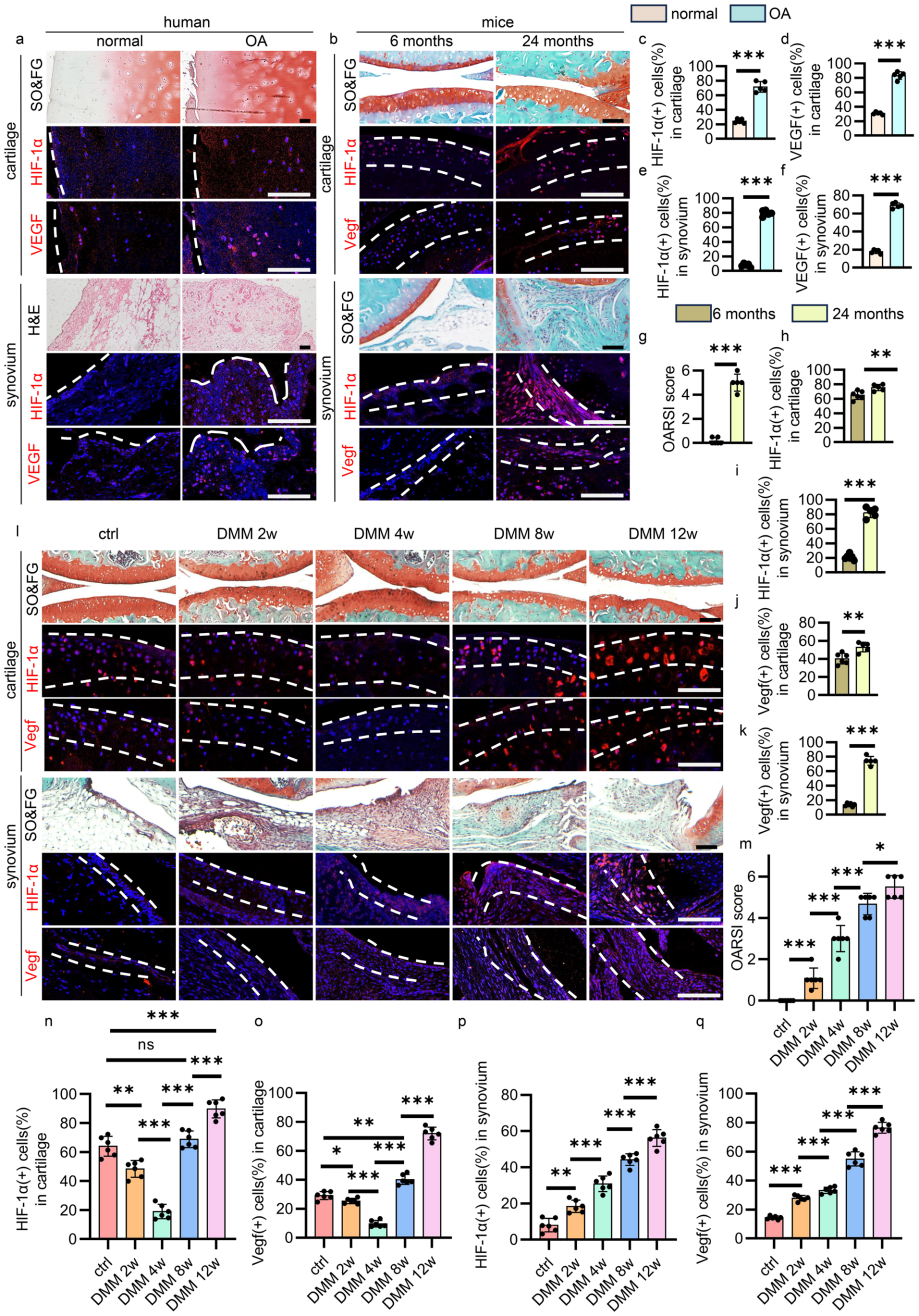
Expression patterns of HIF‐1α and Vegf in different OA models. (a) SO&FG, HE and IF staining of human articular cartilage and synovium sections from the same patient. Scale bar, 100 μm. (b) SO&FG and IF staining of knee joint sections in 6‐month‐old mice (*n* = 6 biological replicates) and 24‐month‐old mice (*n* = 5 biological replicates) groups showing the expression of HIF‐1α and Vegf in articular cartilage and synovium. Scale bar, 100 μm. (c–f) Quantitative data of expression of HIF‐1α (c) and Vegf (d) in human articular cartilage, and HIF‐1α (e) and Vegf (f) in and synovium. *n* = 5 regional biological replicates derived from distinct macroscopic areas of a single patient's joint. g. OARSI score was performed using SO&FG sections of mice. *n* = 6 biological replicates. (h–k) Quantitative data of expression of HIF‐1α in articular cartilage (h) and synovium (i), and Vegf in articular cartilage (j) and synovium (k) of mice. (l) SO&FG and IF staining of serial knee joint sections at 2, 4, 8, and 12 weeks post‐DMM surgery, alongside sham‐operated controls, showing the expression of HIF‐1α and Vegf in articular cartilage and synovium. *n* = 6 biological replicates. Scale bar, 100 μm. (m) OARSI score was performed using SO&FG sections. (n–q) Quantitative data of expression of HIF‐1α and Vegf in articular cartilage (n, o) and synovium (p, q). *n* = 6 biological replicates. Quantitative data are shown as mean ± SD. ns, no statistical significance, *p < 0.05, **p < 0.01, ***p < 0.001.

To establish the clinical relevance of hypoxia signaling in OA, we first collected paired osteochondral and synovial samples from the same OA patients undergoing joint replacement surgery. Within joint, relatively preserved cartilage and synovium were harvested as the normal group, while obviously worn cartilage and hyperplastic synovium from inflamed regions were collected as the OA group. Histological assessments using SO&FG and H&E staining confirmed significant pathological divergence between the two groups (Figure 1a). In particular, the OA group was characterized by evident wear and fibrillation of the superficial zone, accompanied by marked proteoglycan depletion and synovial hyper‐inflammation. Immunofluorescence (IF) staining revealed that HIF‐1α expression was significantly elevated in both the degraded cartilage and the inflamed synovium compared to their respective intact counterparts (Figure 1a,c,e). Notably, the expression of its downstream angiogenic effector, VEGF, was also concurrently upregulated in these OA‐affected tissues (Figure 1a,d,f).

We also evaluated knee joint sections from 6‐ and 24‐month‐old mice. SO&FG staining revealed evident osteoarthritic changes in the older mice, which were quantitatively confirmed by significantly higher Osteoarthritis Research Society International (OARSI) scores in the 24‐month‐old group compared to the 6‐month‐old group (Figure 1b,g). Furthermore, immunofluorescence (IF) staining and subsequent quantitative analysis demonstrated significant changes in the expression levels of both HIF‐1α and Vegf within the articular cartilage and synovium of 24‐month‐old mice (Figure 1b,h–k).

To determine whether similar expression patterns occur in post‐traumatic osteoarthritis (PTOA), 3‐month‐old mice were subjected to DMM surgery, and serial knee joint sections were evaluated at 2, 4, 8, and 12 weeks post‐surgery, alongside sham‐operated controls. SO&FG histological evaluation and OARSI scoring confirmed a progressive worsening of joint destruction over the 12‐week postoperative period (Figure 1l,m). Consistent with the structural degradation observed, IF quantitative analysis revealed distinct expression profiles of HIF‐1α and Vegf in both the articular cartilage and synovium across the post‐DMM timeline compared to sham controls (Figure 1l,n–q). Within the avascular articular cartilage, both HIF‐1α and Vegf exhibited a dynamic biphasic response—an initial reduction during the early postoperative phase (2–4 weeks), followed by a significant surge at the later stages of joint degeneration (8–12 weeks). In contrast, the highly vascularized synovium demonstrated a continuous, progressive accumulation of these proteins throughout the disease course (Figure 1l,n–q).

Together, these clinical and in vivo findings highlight distinct, compartment‐specific dynamics of the HIF‐1α/Vegf axis during OA progression. Notably, while the avascular cartilage exhibits a dynamic fluctuation in HIF‐1α and Vegf expression during early disease development followed by a severe late‐stage surge, the highly vascularized synovium demonstrates a continuous, progressive pathological accumulation that mirrors the clinical presentation of human OA. To determine whether the sustained accumulation of HIF‐1α in these distinct joint tissues acts as an active pathogenic driver of joint destruction rather than a mere compensatory response, we next utilized genetically engineered mouse models to enforce sustained HIF‐1α expression.

### 3.2 Chondrocyte‐Specific Overexpression of HIF‐1α Exacerbates PTOA

**FIGURE 2:**
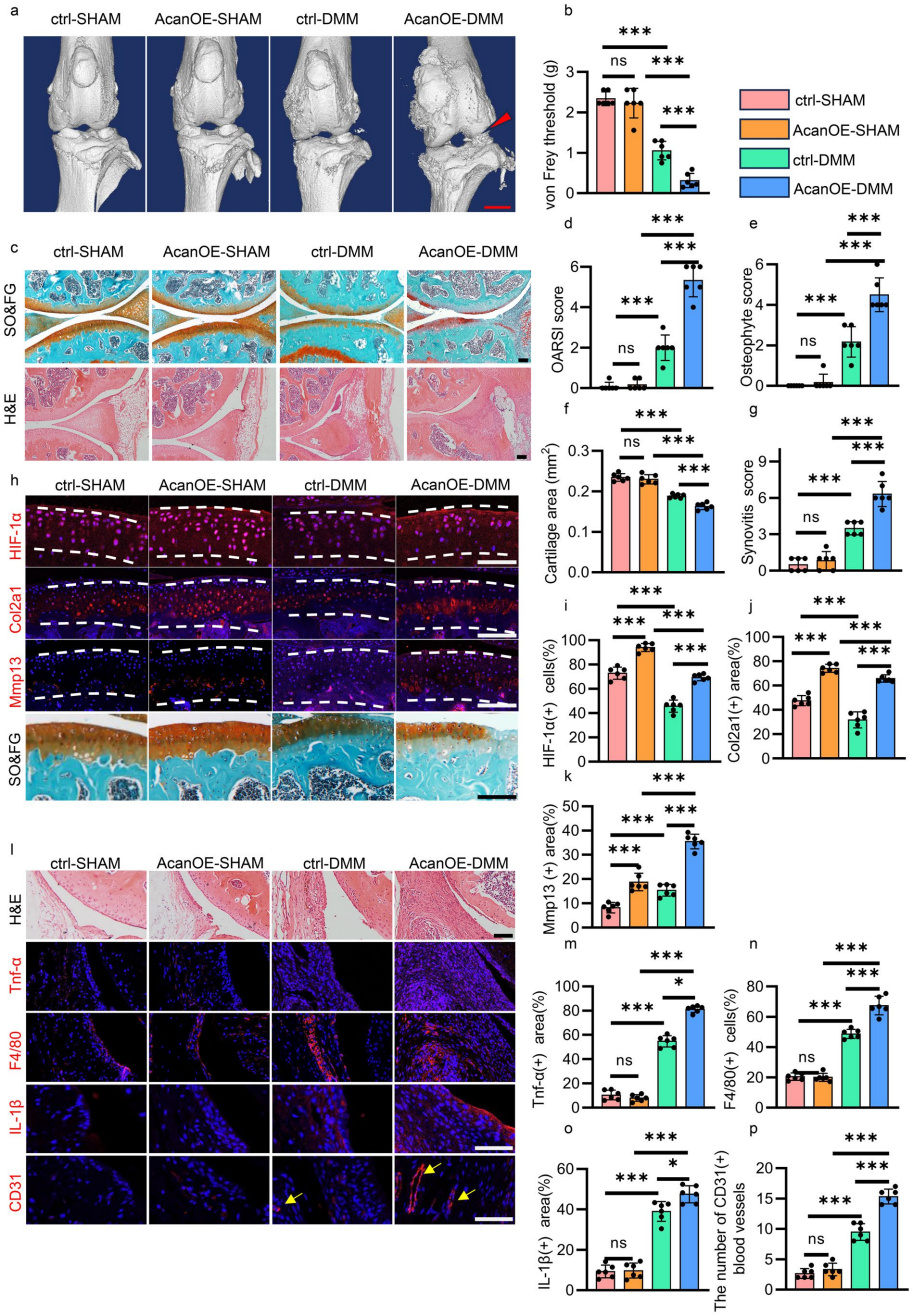
Chondrocyte HIF‐1α overexpression exacerbates PTOA. (a) Representative high‐resolution μCT 3D reconstructions and structural analysis of knee joints at one month post‐DMM surgery. *n* = 8 biological replicates. Scale bar, 1 mm. (b) Quantitative behavioral assessment of mechanical allodynia using the von Frey filament test. *n* = 6 biological replicates. c. Histological evaluation via SO&FG and H&E staining. *n* = 6 biological replicates. Scale bar, 200 μm. (d–g) Quantification of OARSI score (d), osteophyte score (e), cartilage area osteophyte score (f) and synovitis score (g) was performed using histological sections. *n* = 6 biological replicates. (h) IF staining of knee joint sections showing the expression of HIF‐1α, Col2a1 and Mmp13 in cartilage. *n* = 6 biological replicates. Scale bar, 200 μm. (i–k) Quantitative data of expression of HIF‐1α (i), Col2a1 (j) and Mmp13 (k) in cartilage. *n* = 6 biological replicates. (l) H&E and IF staining of knee joint sections showing the expression of Tnf‐α, F4/80, IL‐1β and CD31 in synovium. *n* = 6 biological replicates. Scale bar, 200 μm. (m–p) Quantitative data of expression of Tnf‐α (m), F4/80 (n), IL‐1β (o) and CD31 (p) in synovium. *n* = 6 biological replicates. Quantitative data are shown as mean ± SD. ns, no statistical significance, *p < 0.05, ***p < 0.001.

Based on prior research, we hypothesized that HIF‐1α overexpression may play a role in the initiation and progression of OA. To verify this hypothesis, we induced HIF‐1α overexpression in Aggrecan‐expressing cells at 3‐month‐old *Acan* ^*CreERT2*^; *Hif1αdPA* ^*fl/fl*^ male mice by treating with five daily intraperitoneal (i.p.) injections of tamoxifen (TAM) (hereafter referred to as AcanOE) (Figure S1a,b). One week post‐induction, mice underwent DMM surgery on the right knee to induce mechanical instability, with sham surgery performed on the left knee. At one month post‐surgery, behavioral assessments using the von Frey filament test revealed that DMM significantly enhanced mechanical allodynia. Notably, the AcanOE‐DMM group exhibited markedly greater pain sensitivity compared to the ctrl‐DMM group (Figure 2b). High‐resolution μCT analysis confirmed successful disease induction, with complete medial meniscus wear observed in all DMM‐operated knees. However, the AcanOE‐DMM group displayed exacerbated joint pathology, characterized by enhanced posterior meniscal mineralization and more pronounced osteophyte formation compared to the ctrl‐DMM group (Figure 2a). Histological evaluation via SO&FG staining corroborated the μCT findings. While ctrl‐DMM mice exhibited typical OA‐like structural changes, including superficial cartilage erosion and synovial inflammation, the progression of these lesions was severely accelerated in AcanOE mice (Figure 2c). The AcanOE‐DMM group presented with deeper cartilage fissures, a more extensive loss of cartilage area, massive osteophytes, and the emergence of distinct ossification centers within the synovial tissues (Figure 2d–g). IF staining confirmed the robust, targeted upregulation of HIF‐1α in the cartilage of AcanOE mice (Figure 2h,i). To probe the molecular consequences of this overexpression, we assessed the balance of cartilage matrix metabolism. In the sham‐operated groups, HIF‐1α upregulation in AcanOE mice triggered a paradoxical concurrent increase in both the catabolic marker Mmp13 and the anabolic marker Col2a1 (Figure 2h–k). Following DMM, control mice exhibited the expected metabolic shift toward degradation (decreased Col2a1, increased Mmp13). Strikingly, the AcanOE‐DMM group maintained abnormally high expression levels of both anabolic and catabolic markers compared to the ctrl‐DMM group. This indicates that sustained HIF‐1α activation drives a “metabolic paradox” where compensatory synthetic efforts are overwhelmed by parallel catabolic signaling.

Furthermore, this profound structural and metabolic decline in the AcanOE‐DMM group was accompanied by profound alterations in the broader joint microenvironment. IF analysis revealed a significant surge in the expression of potent pro‐inflammatory cytokines, including IL‐1β and Tnf‐α, within both the degenerating articular cartilage and the surrounding synovial tissues. Concurrently, this hyper‐inflammatory milieu was closely coupled with active immune cell infiltration and pathological tissue remodeling, evidenced by a marked increase in CD31‐positive blood vessels invading the joint tissues alongside a robust accumulation of F4/80‐positive macrophages within the hyperplastic synovial lining (Figure 2l–p). This pathological hypervascularization, combined with the intense recruitment of F4/80^+^ macrophages and heightened cytokine cascade, suggests that chondrocyte HIF‐1α overexpression not only destabilizes intrinsic cartilage matrix homeostasis but also leads to excessive angiogenesis and inflammation within the synovial cavity.

### 3.3 Excessive HIF‐1α Accumulation Is Sufficient to Drive Spontaneous OA

**FIGURE 3:**
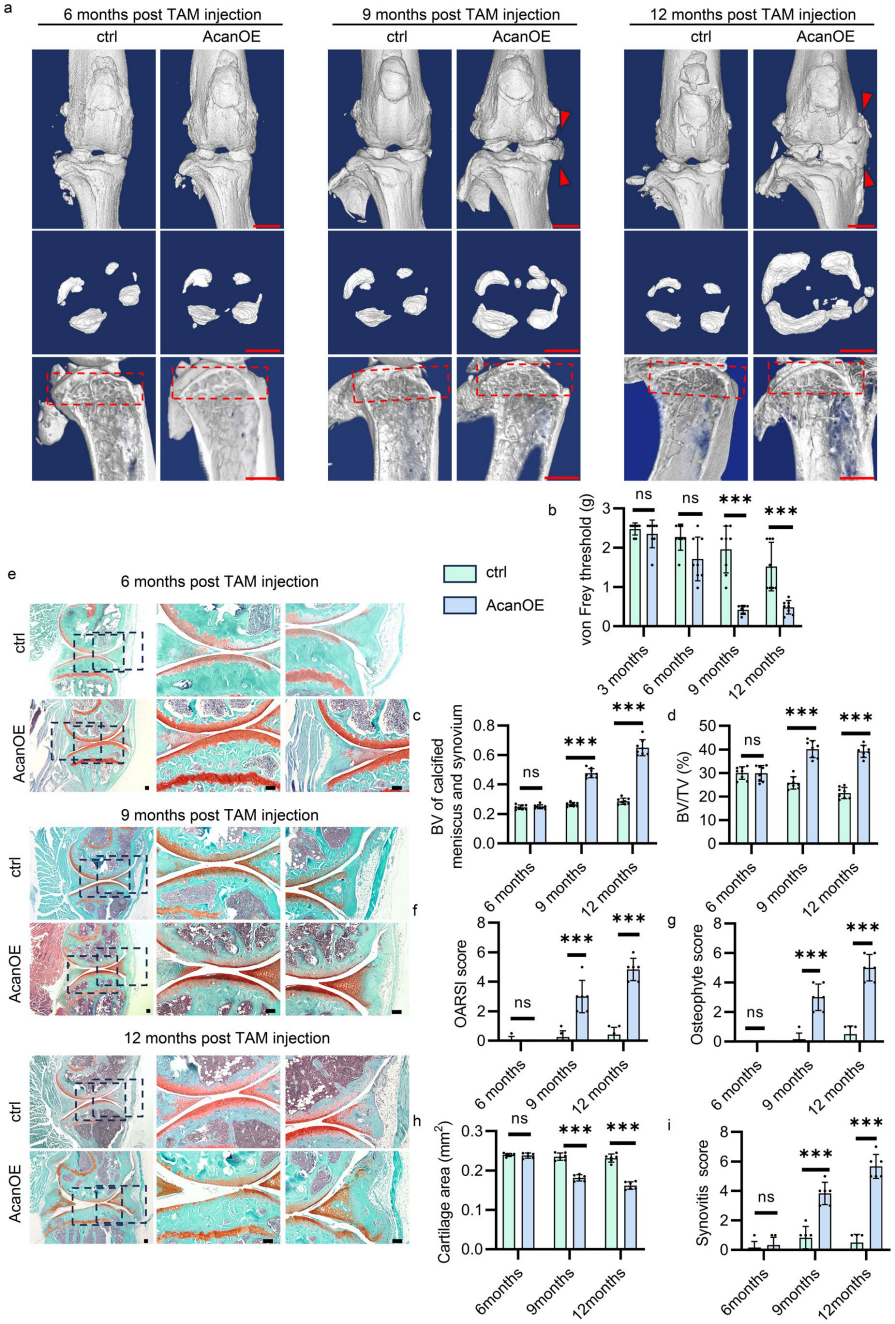
Sustained chondrocyte HIF‐1α accumulation drives spontaneous OA. (a) Representative high‐resolution μCT 3D reconstructions and structural analysis of knee joints of AcanOE mice and their control at 6, 9, and 12 months post‐TAM induction. *n* = 6 biological replicates. Scale bar, 1 mm. (b) Quantitative behavioral assessment of mechanical allodynia using the von Frey filament test at 3, 6, 9, and 12 months post‐TAM injection. *n* = 8 biological replicates. (c) Quantification of BV of calcified meniscus and synovium. *n* = 6 biological replicates. (d) Quantification of BV/TV within the epiphyseal trabecular bone subregion. (e) Histological evaluation via SO&FG staining. Scale bar, 200 μm. *n* = 6 biological replicates. (f–i) Quantification of OARSI score (f), osteophyte score (g), cartilage area (h) and synovitis score (i) was performed using histological sections. *n* = 6 biological replicates. Quantitative data are shown as mean ± SD. ns, no statistical significance, ***p < 0.001.

We next sought to determine whether the uncoupled accumulation of HIF‐1α is sufficient to drive spontaneous joint degeneration in the absence of any mechanical injury. To address this, we conducted a long‐term longitudinal assessment of unchallenged AcanOE mice and their control littermates, performing behavioral and structural analyses at 3‐month intervals following TAM induction. At 6 months post‐induction, both behavioral and structural parameters remained largely stable. The AcanOE group exhibited no significant changes in mechanical pain sensitivity via the von Frey test, and μCT analysis detected no obvious joint abnormalities compared to controls (Figure 3a–d). Consistent with these radiological findings, SO&FG staining revealed intact articular cartilage with no significant OA‐like structural changes at this stage (Figure 3e).

However, a distinct pathological inflection point occurred at 9 months post‐induction. Unchallenged AcanOE mice began to exhibit a significant increase in mechanical allodynia. Concurrent μCT scans revealed the spontaneous emergence of osteophytes and a notable increase in the bone volume (BV) of meniscal mineralization compared to the control group, which was accompanied by an early onset of aberrant subchondral bone remodeling in these 9‐month‐old AcanOE mice, as evidenced by a significant elevation in the bone volume fraction (BV/TV) within the epiphyseal trabecular bone subregion (Figure 3a–d). Histological evaluation further confirmed this spontaneous disease onset, demonstrating a severe loss of articular cartilage, elevated OARSI scores, synovial inflammation, and the formation of small osteophytes in AcanOE mice (Figure 3e–i).

By 12 months post‐induction, the spontaneous joint destruction in the AcanOE group had progressed to a severe, end‐stage osteoarthritic phenotype. Pain sensitivity remained significantly elevated, while μCT and histological analyses revealed substantially increased osteophyte formation, extensive meniscal mineralization, advanced subchondral bone sclerosis and characteristic full‐thickness cartilage erosion (Figure 3a–i). Taken together, these longitudinal data strongly suggest that sustained, chondrocyte‐specific HIF‐1α overexpression is not merely an exacerbating factor, but a primary, sufficient driver of spontaneous OA pathogenesis.

### 3.4 Continuous HIF‐1α Accumulation Disrupts the Hypoxic Niche and Drives Cartilage Metabolic Disorder

**FIGURE 4:**
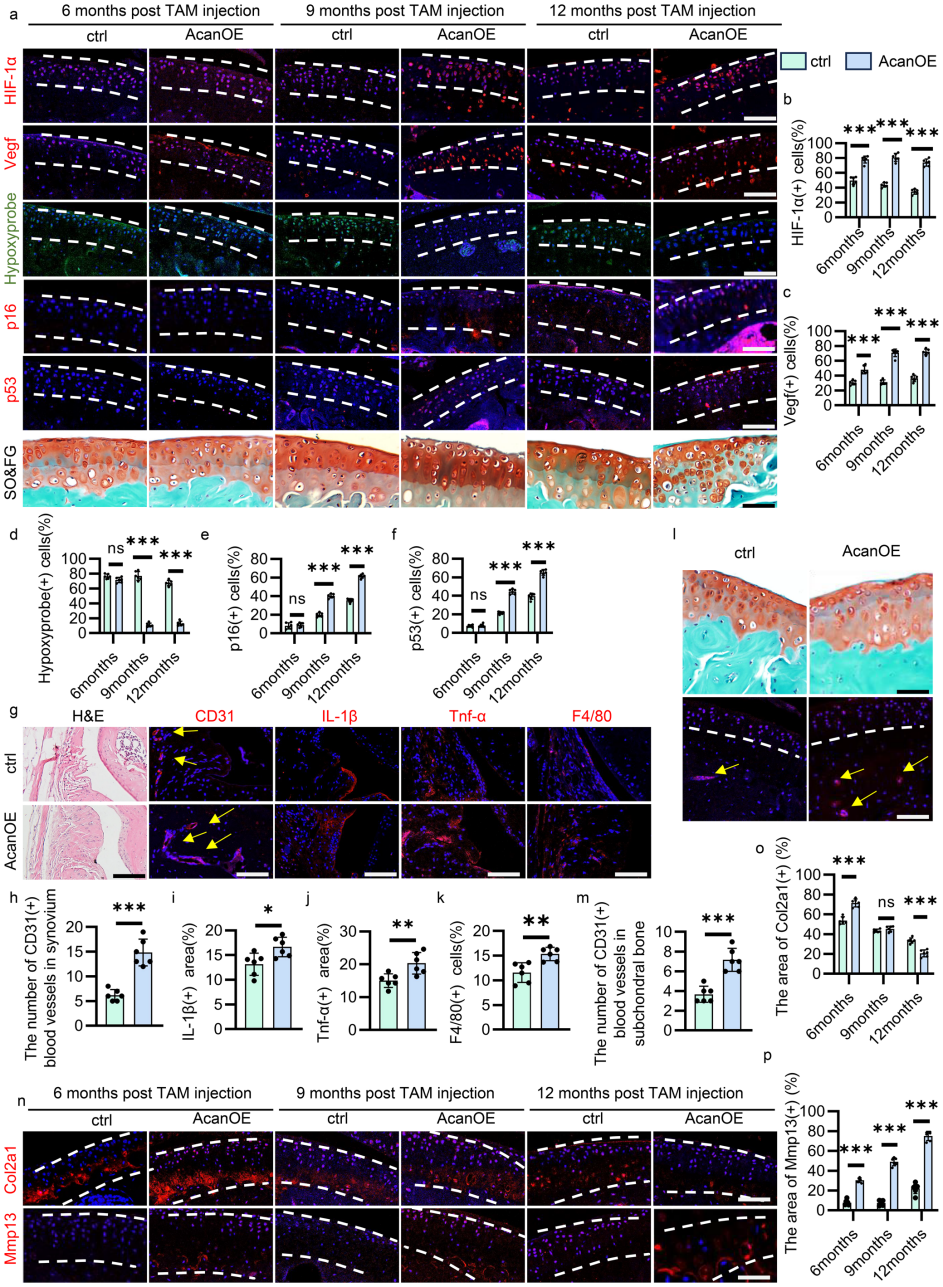
HIF‐1α accumulation disrupts the hypoxic niche and drives cartilage metabolic disorder. (a–f) IF staining of HIF‐1α, Vegf Hypoxyprobe, p16 and p53 and quantitative analysis of HIF‐1α (b), Vegf (c), Hypoxyprobe (d), p16 (e) and p53 (f) in the articular cartilage of AcanOE mice and their controls at 6, 9, and 12 months post‐TAM injection. *n* = 6 biological replicates. Scale bar, 200 μm. (g–k) IF staining and quantitative analysis of CD31 (h), IL‐1β (i), Tnf‐α (j) and F4/80 (k) in synovium. (l, m) IF staining and quantitative analysis of CD31 in subchondral bone. *n* = 6 biological replicates. Scale bar, 200 μm. (n–p) IF staining and quantitative analysis of Col2a1 (o) and Mmp13 (p) in cartilage. *n* = 6 biological replicates. Scale bar, 200 μm. Quantitative data are shown as mean ± SD. ns, no statistical significance, *p < 0.05, ***p < 0.001.

IF staining confirmed sustained overexpression of HIF‐1α and its downstream target Vegf in the AcanOE group at 6, 9, and 12 months post‐TAM injection (Figure 4a–c). Interestingly, hypoxyprobe analysis unveiled a temporal eradication of the physiological hypoxic microenvironment that closely corresponded with the metabolic collapse. While articular chondrocytes in both groups maintained normal, robust hypoxic signatures at 6 months, the AcanOE group exhibited a significant depletion of hypoxic cells by 9 months—the exact time point when spontaneous structural damage occurred. By 12 months, the physiological hypoxic signal within the AcanOE cartilage was essentially abolished (Figure 4a,d).

To investigate whether the chronic metabolic disorder driven by sustained HIF‐1α accumulation eventually precipitates cellular senescence within the articular cartilage, we performed longitudinal immunofluorescence staining for the canonical senescence markers p16INK4a (p16) and p53 at multiple time points post‐TAM induction. At 6 months post‐induction, no discernible differences in the expression of either p16 or p53 were observed between the AcanOE group and their controls, indicating that the cartilage remnants retained a nonsenescent state during this phase (Figure 4a,e,f). However, a distinct pathological shift occurred at 9 months post‐induction, where the AcanOE cartilage exhibited a significant and robust increase in both p16‐ and p53‐positive chondrocytes compared to the controls (Figure 4a,e,f). This senescent phenotype became progressively exacerbated by 12 months post‐induction, demonstrated by a further profound surge in p16 and p53 accumulation throughout the degenerating cartilage layers (Figure 4a,e,f). Together, these time‐course findings reveal a clear spatiotemporal trajectory, demonstrating that while short‐term HIF‐1α elevation is cellularly tolerated, chronic and excessive HIF‐1α accumulation over time acts as a potent trigger that inevitably drives chondrocyte senescence, accelerating the catastrophic collapse of the articular niche in advanced OA stage.

We next investigated whether this severe metabolic shift was mechanistically linked to the physical disruption of the avascular cartilage niche and the induction of a pro‐inflammatory microenvironment. Given the sustained upregulation of Vegf, we evaluated joint vascularization and inflammatory dynamics. CD31 immunostaining at 9 months revealed a striking pathological invasion of blood vessels into the subchondral bone and synovial compartments of AcanOE mice (Figure 4g,h,l,m). Concurrently, IF analysis demonstrated a significant surge in the expression of potent pro‐inflammatory cytokines, including IL‐1β and TNF‐α, within the synovium, alongside a robust accumulation and recruitment of F4/80‐positive macrophages within the hyperplastic synovial lining, highlighting an intense inflammatory response that is intimately coupled with the abnormal angiogenesis (Figure 4g,i–k).

Notably, at 6 months post‐induction—prior to the onset of overt structural OA—we observed the distinct emergence of the “metabolic paradox” initially noted in the DMM model. Chondrocyte HIF‐1α overexpression drove a simultaneous, robust upregulation of both the anabolic marker Col2a1 and the catabolic marker Mmp13 (Figure 4n–p). However, as the disease progressed to the 9‐month inflection point, this fragile hypermetabolic state collapsed. The compensatory upregulation of Col2a1 failed to be maintained, subsiding to levels comparable to the control group, while the expression of Mmp13 was further and potently induced. By 12 months, this temporal uncoupling of anabolism and catabolism culminated in a profound matrix degradation profile that was overwhelmingly dominated by Mmp13.

Collectively, these temporal data demonstrate that continuous HIF‐1α activation actively dismantles the joint ecosystem. The HIF‐1α/VEGF‐driven pathological angiogenesis physically eradicates the essential hypoxic niche and triggers profound synovial inflammation, forcing a fatal metabolic shift. Crucially, while this persistent metabolic stress is cellularly tolerated during the early subclinical phase, it inevitably precipitates widespread cellular senescence in advanced stages, as evidenced by the progressive and profound accumulation of p16 and p53 within the aging chondrocytes. This cascade forces a fatal structural and cellular shift, irreversibly tipping the cartilage from a state of frustrated repair into overwhelming, chronic degeneration and catastrophic articular niche collapse.

### 3.5 Sustained HIF‐1α Activation in Prg4‐Lineage Cells Drives Late‐Onset Spontaneous OA and Sensitizes the Joint to Trauma

Having demonstrated that intrinsic HIF‐1α accumulation in the whole layer of cartilage actively drives profound joint destruction, we next investigated the specific pathological contribution of sustained HIF‐1α activation within the superficial cartilage compartments. To achieve this, we utilized the *Prg4* ^*GFPCreERT2*^ driver to generate *Prg4* ^*GFPCreERT2*^; *Hif1αdPA* ^*fl/fl*^ mice (hereafter referred to as Prg4OE) and longitudinally monitored their spontaneous disease progression following TAM induction (Figure S2a,b). IF analysis revealed clear compartment‐specific accumulation patterns (Figure S2c–e). In the AcanOE model, sustained HIF‐1α expression was distributed across the full‐thickness articular cartilage and was largely restricted to the superficial layer of the synovium. In contrast, within the Prg4OE model, robust HIF‐1α overexpression was predominantly localized to the superficial zone of the cartilage and extended broadly across the entire synovial lining. This distinct spatial divergence provides a precise genetic tool to specifically investigate the pathological contribution of sustained HIF‐1α activation within the highly vascularized synovial and superficial cartilage compartments.

**FIGURE 5:**
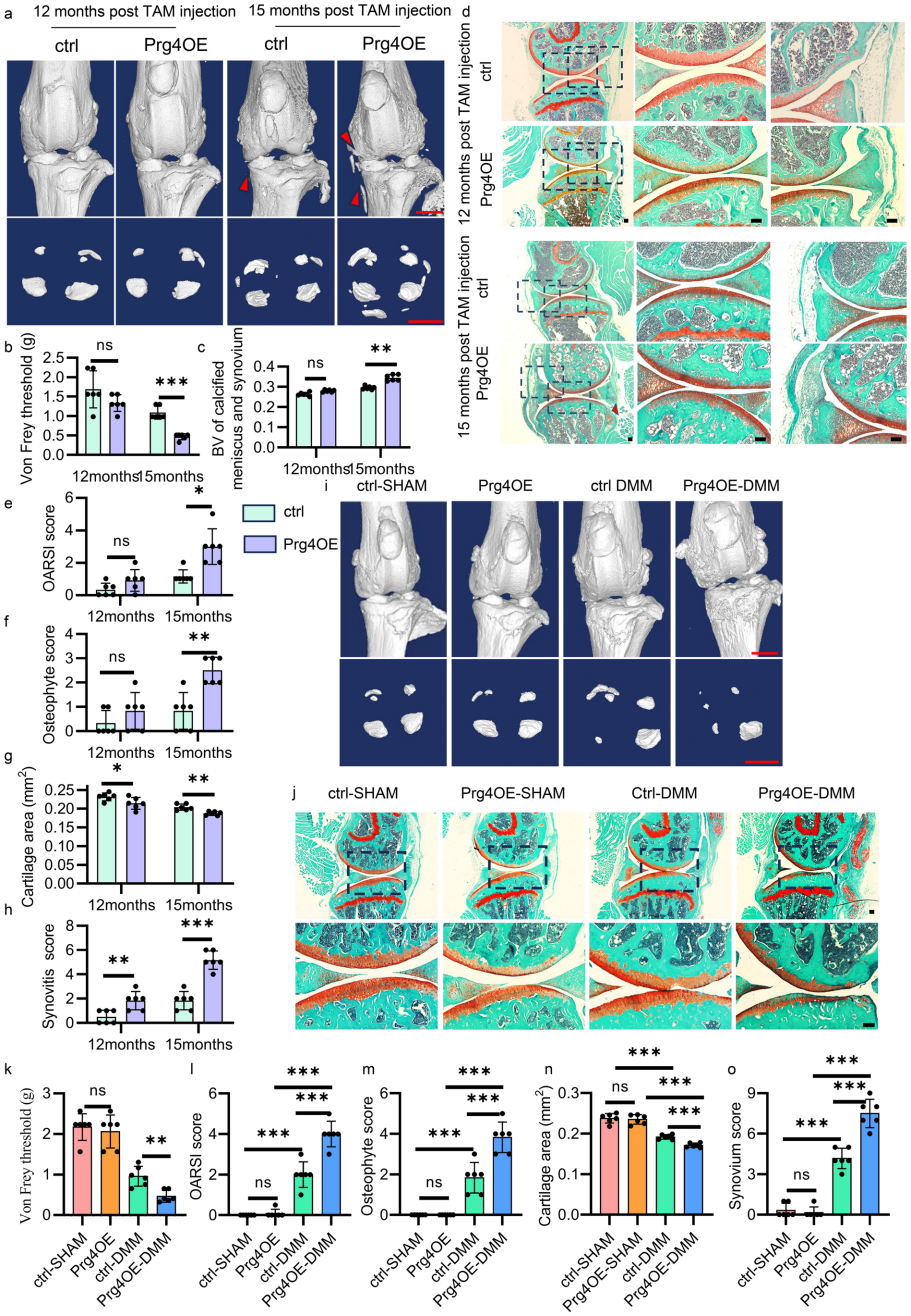
HIF‐1α accumulation in Prg4‐lineage cells drives late‐onset OA and trauma sensitization. (a) Representative high‐resolution μCT 3D reconstructions and structural analysis of knee joints of Prg4OE mice and their control 12 and 15 months post‐TAM induction. *n* = 6 biological replicates. Scale bar, 1 mm. (b) Quantitative behavioral assessment of mechanical allodynia using the von Frey filament test at 12 and 15 months post‐TAM injection. *n* = 6 biological replicates. (c) Quantification of BV of calcified meniscus and synovium. *n* = 6 biological replicates. (d) Histological evaluation via SO&FG staining. Scale bar, 200 μm. (e–h) Quantification of OARSI score (e), cartilage area (f), osteophyte score (g) and synovitis score (h) was performed using histological sections of Prg4OE mice and their control. *n* = 6 biological replicates. (i–k) Radiological (i), SO&FG staining (j), and behavioral (k) evaluations of the Prg4OE‐DMM model. *n* = 6 biological replicates. Scale bar, 1 mm (i). Scale bar, 200 μm (j). (l–o) Quantification of OARSI score (l), osteophyte score (m), cartilage area osteophyte score (n) and synovitis score (o) was performed using histological sections of Prg4OE‐DMM model. *n* = 6 biological replicates. Quantitative data are shown as mean ± SD. ns, no statistical significance, *p < 0.05, **p < 0.01, ***p < 0.001.

In contrast to the relatively rapid pathological onset observed in the AcanOE model, Prg4OE mice exhibited a more delayed, late‐onset trajectory of joint degeneration. At 12 months post‐induction, macroscopic and behavioral assessments remained largely unremarkable. The Prg4OE mice showed no significant increase in mechanical pain sensitivity via the von Frey test, and μCT analysis did not detect obvious macroscopic structural abnormalities compared to controls (Figure 5a–c). However, early microscopic manifestations of joint deterioration were already evident at this stage. SO&FG staining revealed a distinct upward shift of the tidemark, a preliminary reduction in the articular cartilage area, and an elevated synovial inflammation score (Figure 5d–h). This discordance suggests that in the Prg4‐expressing niche, microscopic pathological remodeling and inflammatory priming precede overt structural failure.

As the mice aged to 15 months post‐induction, this prolonged microscopic remodeling culminated in full‐blown, symptomatic osteoarthritis. Behavioral testing demonstrated a significant enhancement in mechanical allodynia in the Prg4OE group. Concurrent μCT scans revealed the distinct emergence of osteophytes and meniscal mineralization (Figure 5a–c). Histological evaluation further corroborated this severe joint destruction, displaying characteristic end‐stage OA‐like changes: extensive superficial cartilage erosion, extensive osteophyte formation, significantly heightened synovial inflammation, and a dramatically more pronounced reduction in the total cartilage area (Figure 5d–h).

While the spontaneous joint degeneration driven by Prg4‐lineage cells follows a prolonged, indolent trajectory, we next investigated whether this sustained synovial and superficial inflammatory priming sensitizes the joint to acute mechanical injury. To this end, 2 weeks following TAM induction, Prg4OE and control mice underwent DMM surgery, and joint samples were harvested for evaluation 4 weeks post‐surgery. In stark contrast to the slow 15‐month progression of the spontaneous disease, mechanical instability precipitated a rapid and severe osteoarthritic phenotype in Prg4OE mice within this short timeframe. Behavioral assessments revealed that the Prg4OE‐DMM group exhibited significantly enhanced mechanical allodynia compared to the ctrl‐DMM group (Figure 5k). Furthermore, μCT and SO&FG evaluations demonstrated that mechanical instability synergized significantly with the Prg4‐specific HIF‐1α accumulation (Figure 5i,j). The Prg4OE‐DMM joints displayed markedly exacerbated arthritic lesions, characterized by elevated OARSI scores, massive osteophyte formation, accelerated loss of the articular cartilage area, and dramatically heightened synovial inflammation scores relative to the ctrl‐DMM group (Figure 5l–o).

### 3.6 Synovial HIF‐1α Accumulation Triggers Inflammatory Priming and Suppresses Cartilage Anabolism

**FIGURE 6:**
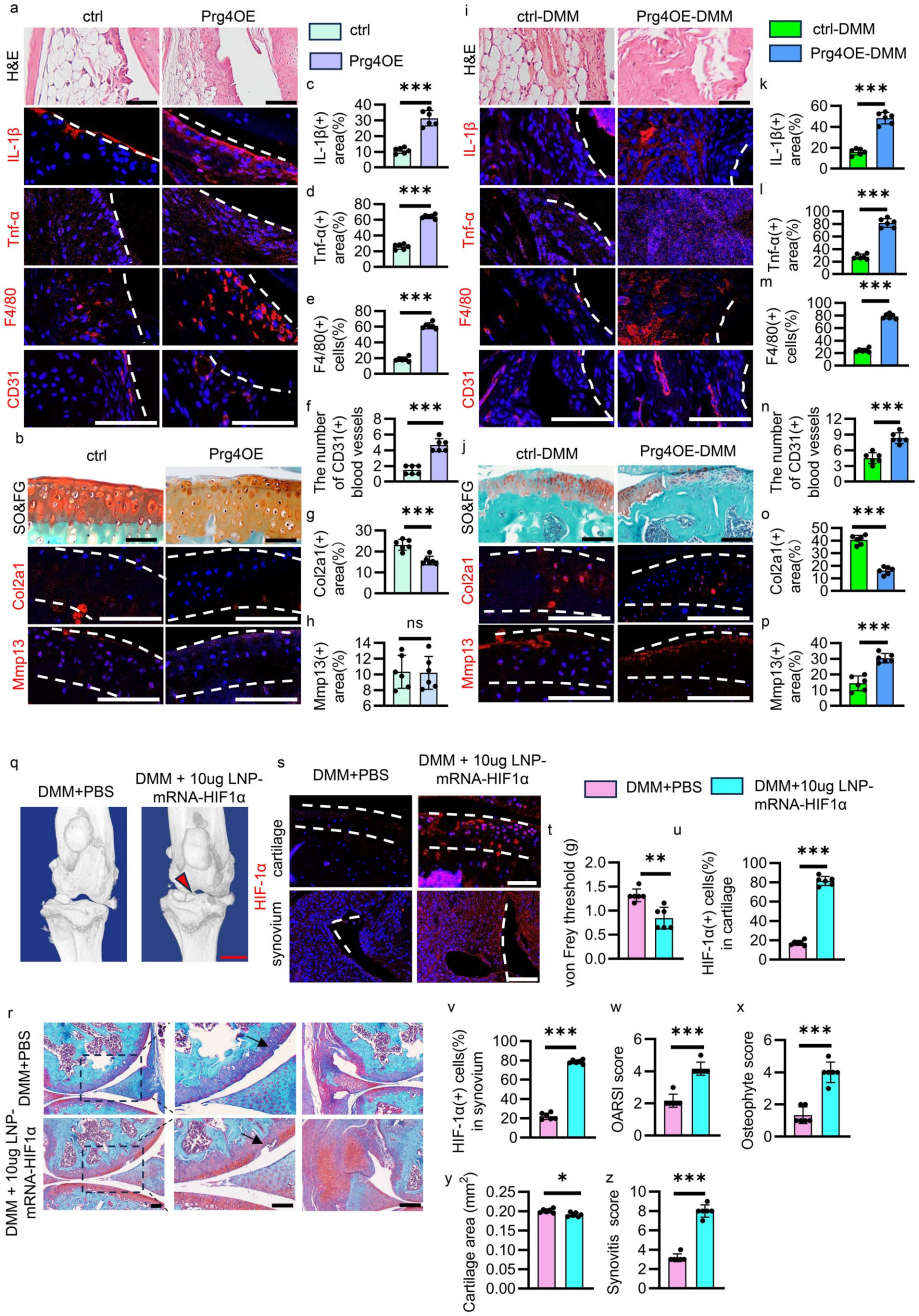
Synovial HIF‐1α triggers inflammatory priming and its whole‐joint activation via LNP‐mRNA exacerbates PTOA. (a, b) H&E and IF staining of IL‐1β, Tnf‐α, F4/80 and CD31 in synovium (a), and SO&FG and IF staining of Col2a1 and Mmp13 in cartilage (b) of Prg4OE mice and control at 12 months post‐TAM injection. *n* = 6 biological replicates. Scale bar, 200 μm. (c–h) Quantitative analysis of IL‐1β (c), Tnf‐α (d), F4/80 (e), CD31 (f), Col2a1 (g) and Mmp13 (h). *n* = 6 biological replicates. (i, j) IF staining of IL‐1β, Tnf‐α, F4/80 and CD31 in synovium (i), and IF staining of Col2a1 and Mmp13 in cartilage (j) of Prg4OE‐DMM mice and control‐DMM mice. *n* = 6 biological replicates. Scale bar, 200 μm. (k–p) Quantitative analysis of IL‐1β (k), Tnf‐α (l), F4/80 (m), CD31 (n), Col2a1 (o) and Mmp13 (p). *n* = 6 biological replicates. (q) Representative high‐resolution μCT 3D reconstructions and structural analysis of knee joints from LNP‐*Hif1α* treated mice and controls. *n* = 6 biological replicates. Scale bar, 1 mm. (r) SO&FG staining evaluations of knee joints sections. *n* = 6 biological replicates. Scale bar, 200 μm. (s) IF staining of HIF‐1α in cartilage and synovium. (t) Behavioral evaluations. (u, v) Quantification of HIF‐1α in cartilage (u) and synovium (v). *n* = 6 biological replicates. Scale bar, 200 μm. (w–z) Quantification of OARSI score (w), osteophyte score (x), cartilage area (y) and synovitis score (z) was performed using histological sections. *n* = 6 biological replicates. Quantitative data are shown as mean ± SD. *p < 0.05, **p < 0.01, ***p < 0.001.

To define the molecular signature underlying this delayed spontaneous degeneration and the acute trauma sensitization observed in the Prg4OE model, we evaluated the joint tissues via IF staining. We specifically focused on the 12‐month time point (where structural integrity was largely maintained but microscopic remodeling had begun—and the catastrophic DMM model). Consistent with the early histological signs of synovitis at 12 months, the Prg4OE synovium displayed a significant surge in the pro‐inflammatory cytokines, including IL‐1β and Tnf‐α, within the hyperplastic synovial tissue. Concurrently, this heightened inflammatory state was tightly coupled with pathological angiogenesis and active immune cell infiltration, as evidenced by a marked increase in CD31‐positive blood vessels alongside a robust accumulation and recruitment of F4/80‐positive macrophages within the synovial compartments (Figure 6a,c–e). Interestingly, within the adjacent articular cartilage, the anabolic marker Col2a1 was already significantly downregulated, even though the catabolic marker Mmp13 remained unchanged relative to controls (Figure 6b,g,h). This distinct molecular profile effectively captures a state of “inflammatory priming and anabolic suppression”—where the HIF‐1α‐driven synovial hypervascularization and inflammation have established a hostile microenvironment that actively blunts cartilage matrix synthesis, rendering the joint functionally vulnerable well before overt structural degradation begins. IF analysis revealed that the rapid structural collapse observed in the Prg4OE‐DMM model was driven by the complete loss of fragile cartilage homeostasis. Following mechanical injury, the synovium exhibited a further catastrophic enhancement of the local inflammatory and vascular cascade. IF analysis revealed a significant surge in the expression of potent pro‐inflammatory cytokines, including IL‐1β and TNFα, which was accompanied by a massive invasion of CD31‐positive blood vessels alongside a robust, dense accumulation of F4/80‐positive macrophages within the hyperplastic synovial compartments (Figure 6i,k–n). Concurrently, the previously observed anabolic suppression within the cartilage was now accompanied by a profound catabolic surge. The Prg4OE‐DMM cartilage displayed a sustained, significant downregulation of Col2a1 coupled with a robust, pathological induction of Mmp13 expression (Figure 6j,o,p).

Taken together, these molecular data demonstrate a striking spatial dichotomy in OA pathogenesis. Distinct from the rapid structural collapse driven by intrinsic metabolic failure in the deep‐zone cartilage (the AcanOE model), uncoupled HIF‐1α accumulation in the synovial and superficial niche acts primarily as a microenvironmental disease amplifier. It initiates a prolonged phase of hypervascular inflammatory priming that actively blunts cartilage anabolism, eventually forcing late‐onset joint failure. Moreover, it critically lowers the threshold for trauma‐induced destruction by priming the anabolically suppressed cartilage for a massive catabolic outburst upon mechanical stress.

### 3.7 Transient Whole‐Joint HIF‐1α Activation via LNP‐mRNA Exacerbates PTOA

To validate our genetic findings and exclude potential developmental artifacts of the Cre‐loxP systems, we tested whether acute, exogenous induction of HIF‐1α is sufficient to aggravate OA progression, employing a highly translational LNP delivery system. Following DMM surgery, wild‐type mice received an intra‐articular injection of LNP encapsulated with *Hif1α* mRNA (LNP‐*Hif1α*). μCT analysis revealed the distinct emergence of osteophytes and meniscal mineralization (Figure 6q). Behavioral testing demonstrated a significant enhancement in mechanical allodynia in the LNP‐*Hif1α* treated mice (Figure 6t). IF staining confirmed the successful, widespread delivery and translation of the mRNA‐*Hif1α*, revealing a robust upregulation of HIF‐1α protein expression within both the articular cartilage and the synovial compartments (Figure 6s,u,v). Consistent with the catastrophic phenotypes observed in our compartment‐specific transgenic models, SO&FG histological evaluation demonstrated that this transient, whole‐joint HIF‐1α over‐accumulation dramatically accelerated post‐traumatic joint destruction. The LNP‐*Hif1α* treated joints exhibited significantly enhanced cartilage erosion, matrix loss, and synovial inflammation compared to controls (Figure 6r,w–z). Collectively, this pharmacological proof‐of‐concept firmly demonstrates that excessive HIF‐1α accumulation—whether driven by intrinsic genetic mutations or acute exogenous stimuli—functions as a broad pathogenic amplifier across the entire joint microenvironment.

## 4 Discussion

The precise role of HIF‐1α in articular biology has long been an enigma, often viewed paradoxically as either an indispensable survival factor or a destructive disease mediator. In this study, we provide compelling in vivo evidence that offers critical insights to reconcile this controversy. We establish that sustained, uncoupled HIF‐1α accumulation is not a mere byproduct of joint degeneration, nor a failed compensatory shield, but a potent, compartment‐specific pathogenic driver of OA. By employing temporally controlled genetic models and highly translational LNP‐mRNA delivery, we reveal that chronic HIF‐1α activation actively dismantles the whole‐joint ecosystem through two distinct but synergistic mechanisms: the physical eradication of the hypoxic cartilage niche via pathological angiogenesis and the induction of a profound “inflammatory priming” state within the synovium and superficial cartilage.

To reconcile our findings with the existing dogma, it is critical to directly compare our model of sustained HIF‐1α accumulation with previous loss‐of‐function studies. Historically, the protective role of HIF‐1α was firmly established by conditional knockout models, such as those demonstrating that genetic deletion of *Hif1a* (Bouaziz et al. 2016) accelerates cartilage destruction and exacerbates OA. These studies confirmed that baseline HIF‐1α is an indispensable survival factor required for chondrocyte maintenance under physiological hypoxia. However, our findings challenge the subsequent assumption that trauma‐induced or age‐related upregulation of HIF‐1α is a successful compensatory response. We theorize a fundamental mechanistic divergence between transient physiological signaling and sustained, uncoupled accumulation. Transient physiological HIF‐1α activation operates within strict regulatory feedback loops, safely maintaining hypoxia‐adapted metabolism and matrix synthesis without disrupting the surrounding microenvironment. In contrast, the sustained, uncoupled accumulation observed in late‐stage OA—and strictly mimicked by our degradation‐resistant genetic models—bypasses these essential regulatory checkpoints. This unremitting signaling shifts HIF‐1α from a physiological protector to a potent metabolic and proteotoxic stressor. It forces chondrocytes into a “metabolic paradox” of simultaneous anabolic and catabolic overdrive, while chronically driving downstream Vegf expression to physically dismantle the avascular cartilage sanctuary via pathological angiogenesis. Thus, while baseline HIF‐1α is essential for survival, its continuous, uncoupled accumulation acts as an active driver of toxicity and joint collapse.

Focusing first on the deep‐zone cartilage, our longitudinal analysis captures the precise molecular transition of the “metabolic paradox”—a phenomenon where compensatory synthetic attempts are ultimately overwhelmed by degradation pathways. In the early stages of AcanOE pathology, we observed a simultaneous surge in both anabolic (Col2a1) and catabolic (Mmp13) markers. This reflects a state of “frustrated repair,” where chondrocytes desperately attempt to rebuild the matrix under HIF‐1α stimulation, yet are simultaneously forced into a hypertrophic‐like, degradative trajectory (van der Kraan and van den Berg 2012). As the disease progressed to the structural inflection point (9–12 months), this fragile hypermetabolic state collapsed, culminating in an irreversible catabolic dominance. This dynamic clearly illustrates that uncoupled HIF‐1α signaling pushes chondrocytes past their metabolic tipping point.

A growing body of evidence underlines that cellular senescence is not merely a bystander of joint aging, but an active enzymatic and metabolic driver of OA pathogenesis (Jeon et al. 2017). In the present study, our longitudinal analysis of nonoperated *AcanOE* mice revealed a distinct, time‐dependent trajectory of chondrocyte senescence characterized by the progressive accumulation of p16INK4a and p53 starting at 9 months and further exacerbating by 12 months post‐induction. This clear chronological correlation strongly suggests that while acute or short‐term hypoxic signaling via HIF‐1α acts as a physiological survival mechanism, its chronic and unremitting hyperactivation inevitably acts as a proteotoxic or metabolic stressor that accelerates cellular aging within the articular niche.

In stark contrast to the rapid structural destruction seen in the deep cartilage, our Prg4OE model unveils a highly novel, delayed disease trajectory orchestrated by the superficial and synovial compartments. Sustained HIF‐1α activation within this highly vascularized niche did not cause immediate structural failure, but rather induced a prolonged, indolent phase of “inflammatory priming and anabolic suppression.” Driven by sustained IL‐1β accumulation, this hostile microenvironment actively blunted cartilage matrix synthesis (Col2a1 downregulation) long before macroscopic damage occurred. Furthermore, the temporal dissociation observed in our Prg4OE model provides crucial mechanistic insight into the well‐documented clinical phenomenon of pain‐structure discordance in OA patients. At 12 months post‐induction, these mice exhibited significant mechanical allodynia and elevated synovitis scores, despite the fact that the histological structural integrity of the articular cartilage remained largely preserved. This strongly suggests that sustained synovial HIF‐1α accumulation acts as an early inflammatory primer. By actively driving aberrant angiogenesis and the release of pro‐inflammatory cytokines within the highly innervated synovial and capsular compartments, the continuous HIF‐1α cascade mediates symptomatic joint pain long before overt radiological or histological cartilage loss occurs. Consequently, targeting the synovial HIF‐1α axis may offer a critical therapeutic window for managing early‐stage OA pain prior to irreversible structural collapse. Crucially, this underlying molecular rewiring significantly lowered the threshold for mechanical stress. Upon DMM surgery, this anabolically suppressed, primed joint exhibited a rapid and overwhelming catabolic outburst, explaining why certain patients with pre‐existing synovial inflammation experience accelerated joint deterioration following minor trauma (Scanzello and Goldring 2012).

Crucially, senescent chondrocytes are known to actively orchestrate whole‐joint degeneration by adopting a specialized hypersecretory state termed the Senescence‐Associated Secretory Phenotype (SASP) (Coryell et al. 2021). Our findings that continuous HIF‐1α accumulation triggers a parallel surge in IL‐1β and TNFα expression—alongside a robust recruitment of F4/80‐positive macrophages into the hyperplastic synovium—perfectly align with the concept of inflammaging. In this context, we hypothesize that the sustained HIF‐1α/Vegf axis not only drives local chondrocyte exit from the cell cycle via p16/p53 pathways but may also promote a hypersecretory state akin to the SASP. Given the concurrent synovial inflammation observed in our AcanOE models, it is plausible to suggest a potential cross‐tissue communication, wherein secreted inflammatory factors might diffuse across the joint space to exacerbate adjacent synovial tissue disruption, further promoting pathological angiogenesis and macrophage‐mediated niche collapse. Collectively, these data point toward a novel molecular link wherein chronic hypoxia‐mimetic stress potentially bridges intrinsic cellular senescence with extrinsic age‐related joint inflammation. This functional crosstalk provides an innovative theoretical framework that warrants further investigation into senolytic or senomorphic interventions for advanced OA.

The translational significance of our findings is strongly reinforced by our transient, whole‐joint HIF‐1α induction via intra‐articular LNP‐mRNA delivery (Kong et al. 2025). By recapitulating the severe joint destruction observed in our genetic models, the LNP‐*Hif1α* experiment effectively rules out potential developmental artifacts of the Cre‐loxP system. More importantly, it provides a crucial pharmacological proof‐of‐concept: forcing HIF‐1α accumulation across the entire articular ecosystem broadly exacerbates post‐traumatic OA. This has profound clinical implications. Currently, targeting the HIF pathway via PHD inhibitors is emerging as an exploratory therapeutic strategy in certain joint diseases (Lyu et al. 2024; Hu et al. 2020; Yao, Wu, et al. 2023). While transient or remote stabilization may offer specific benefits, sustained HIF‐1α activation carries significant risks of inducing pathological angiogenesis and maladaptive metabolic reprogramming (Li and Ramli 2025). Our findings provide direct in vivo evidence supporting this critical caveat within the spatiotemporal context of the articular joint. We demonstrate that continuous, local HIF‐1α accumulation—rather than providing chondroprotection—severely disrupts the avascular cartilage niche and initiates insidious synovial inflammatory priming. Therefore, while systemic HIF‐1α stabilization might benefit specific immune‐mediated conditions, our data raise a strong cautionary note against blanket HIF‐1α activation (such as intra‐articular PHD inhibition) for advanced OA. Instead, for OA joints already exhibiting profound metabolic dysregulation, promoting the targeted degradation or inhibition of HIF‐1α represents a highly promising and rational therapeutic direction.

While this study comprehensively delineates the spatiotemporal pathology of HIF‐1α, certain limitations remain. Future investigations should employ advanced metabolomics (e.g., ^13^C‐glucose tracing) to pinpoint the exact metabolic flux alterations driving the “metabolic paradox” and further dissect the specific downstream effectors mediating the cartilage‐synovium crosstalk. In conclusion, we establish HIF‐1α as a critical molecular rheostat in joint homeostasis. Its sustained dysregulation acts as a spatiotemporally dynamic, broad disease amplifier, and recognizing this paradigm shift is essential for the development of next‐generation, precision disease‐modifying OA drugs.

## Author Contributions

Study design: W.G., C.T., Q.Y., C.W., and G.X. Conducting study, data collection and analyses: W.G., C.T., R.L., X.W., J.L., X.G., J.H., M.Q., M.C., F.W., D.C., L.L., and P.W. Data interpretation: W.G., C.T., and C.W. Drafting the manuscript: W.G., C.W., and G.X. W.G., C.W., and G.X. take the responsibility for the integrity of the data analysis.

## Funding

The authors acknowledge the assistance of Core Research Facilities of Southern University of Science and Technology. This work was supported, in part, by the Shenzhen Medical Research Funds (B2504003, B2402033, E250200210), the National Natural Science Foundation of China Grants (82250710175, 82430078, 82261160395, 82230081), the Noncommunicable Chronic Diseases‐National Science and Technology Major Project (2025ZD0550400), the Science and Technology Innovation Commission of Shenzhen Municipal Government Grants (ZDSYS20140509142721429), and the Guangdong Provincial Science and Technology Innovation Council Grant (2017B030301018).

## Ethics Statement

Southern University of Science and Technology, China (SUSTech‐SL2025090803). The Seventh Affiliated Hospital of Sun Yat‐sen University, Shenzhen, China (KY‐2026‐178‐01).

## Conflicts of Interest

The authors declare no conflicts of interest.

## Supporting information


**Figure S1:** The breeding of Acan‐CreERT2; *Hif1αdPAfl/fl* mouse. (a) The breeding scheme. (b) PCR genotyping using tail DNA. *Aggrecan‐Cre*(+), 650 bp. *Hif1αdPA‐flox*, 290 bp; *Hif1α* WT, ~426 bp.
**Figure S2:** The breeding of Prg4‐CreERT2/+; *Hif1αdPAfl/fl* mouse.

## Data Availability

All data presented in this study are available from the corresponding author upon reasonable request.
