# Supplementary material for: Sustained Hypoxia‐Inducible Factor 1‐Alpha Accumulation Disrupts the Articular Niche to Promote Osteoarthritis Pathogenesis
Source: Aging Cell. 2026 Jun 23;25(7):e70606. doi: 10.1111/acel.70606 (PMC13290656; doi:10.1111/acel.70606)
Supplement: Supplementary file 1 — Figure S1: The breeding of Acan‐CreERT2; Hif1αdPAfl/fl mouse. (a) The breeding scheme. (b) PCR genotyping using tail DNA. Aggrecan‐Cre(+), 650 bp. Hif1αdPA‐flox, 290 bp; Hif1α WT, ~426 bp. Figure S2: The breeding of Prg4‐CreERT2/+; Hif1αdPAfl/fl mouse. [file ACEL-25-e70606-s001.docx]

**Histology and immunostaining assays**

Mouse knee joints were fixed in 4% paraformaldehyde (PFA) for 24 hours at 4°C, followed by decalcification in 10% EDTA (pH 7.2) for 21 days. The tissues were then dehydrated through a graded ethanol series, cleared in xylene, and embedded in paraffin^31,35^. Serial sections (5 μm thickness) were prepared and stained with Hematoxylin and Eosin (H&E; Thermo Fisher, #7211 & 7111) or Safranin O and Fast Green (SO&FG; Solarbio, #G1371) according to the manufacturers' protocols for general morphological and cartilage-specific evaluation.

For IF staining, tissue sections underwent the same initial preparation steps as for histological analysis, including deparaffinization, rehydration, and antigen retrieval. After antigen retrieval, sections were permeabilized with 0.1% Triton X-100 in PBS for 15 minutes and then blocked for 1 hour at room temperature using a commercial blocking buffer (QuickBlock™ from Beyotime) to prevent non-specific antibody binding. The sections were then incubated with primary antibodies against HIF-1α(NOVUS, NB100-479, 1:200), Glut1(Abcam, ag982, 1:100), Col2a1(Col2a1, ab34712, 1:200), Mmp13(Abcam, lot:ab51072,1:200), IL-1β(CST, 31202, 1:200) or CD31(Abcam , 28364, 1:200) diluted in the blocking buffer at 4°C overnight. Following extensive washes, the sections were incubated with species-specific secondary antibodies conjugated to Alexa Fluor 568, with all steps performed in the dark to prevent fluorophore bleaching. Cell nuclei were counterstained with DAPI (1 µg/mL) for 5-10 minutes. After a final series of washes, the sections were mounted using an anti-fade mounting medium. Stained sections were examined and imaged using a ZEISS LSM 980 confocal microscope. Image analysis and fluorescence intensity quantification were performed using ImageJ software. All histological scoring and quantitative analyses for IF were conducted in a double-blinded manner to ensure objectivity.

For hypoxia evaluation, mice received an intraperitoneal injection of pimonidazole hydrochloride (60 mg/kg, Hypoxyprobe, HP2-100Kit) 24 hours before sacrifice. Joint sections were then incubated with a mouse monoclonal anti-pimonidazole antibody (1:200) following standard IF protocols. The reduction of hypoxic signatures was quantified as the percentage of Hypoxyprobe-positive chondrocytes per total cells.

**LNP-mRNA**

In vitro transcription and purification of mRNA, in vitro transcription (IVT) of mRNA was performed using T7 RNA polymerase with the promoter sequence 5'-ATTTAGGTGACACTATAG-3'. mRNA was synthesized from linearized plasmid DNA, with incorporation of a 5' cap and a 3' poly-A tail. The reaction contained RNase-free water, NTPs, Cap1, and N1-UTP, followed by addition of 10 × Transcription Buffer and 5µg DNA template. RNase inhibitor, yeast inorganic pyrophosphatase, and T7 RNA polymerase were included, and the mixture was incubated at 37 °C for 2-3 h. DNase I was then added to digest template DNA and mRNA was purified using the Monarch RNA Purification Kit. mRNA concentration was measured with a NanoDrop 2000c UV-Vis spectrophotometer.

LNP-mRNA preparation and quality control, LNPs were formulated by dissolving the ionizable lipid (DLin-MC3-DMA), cholesterol, helper lipid (DSPC), and PEG-lipid in ethanol (molar ratio 50:38.5:10:1.5; total lipid 10 mg/mL). mRNA was diluted in 50 mM sodium citrate (pH = 4) to 0.2 mg/mL. The organic and aqueous phases were combined at a 1:3 (v/v) ratio at 12 mL/min using a microfluidic mixer device. The LNP-mRNA mixture was immediately diluted with sterile PBS (10 mM, pH = 7.2) and concentrated/washed using Amicon® Ultra-15 centrifugal filters (10kDa cutoff), spinning at 4,000 × g, 4 °C for 15-30 min. The PBS wash was repeated three times to reach a final mRNA concentration of 1 mg/mL. Formulations were stored at 4 °C and used within one week. Encapsulation efficiency was quantified using the RiboGreen RNA Assay Kit (Thermo Fisher Scientific). Particle size and distribution were measured on a Malvern Zetasizer, and zeta potential was determined using a Zetasizer Nano ZS.
